# Concurrent function of high-strength dry carbon fiber as resistive heating element and thermistor in ambient air

**DOI:** 10.1016/j.heliyon.2022.e12051

**Published:** 2022-11-30

**Authors:** Danial Forouhar, Jackrit Suthakorn

**Affiliations:** Department of Biomedical Engineering, Center for Biomedical and Robotics Technology (BART LAB), Faculty of Engineering, Mahidol University, Salaya, Thailand

**Keywords:** Carbon fiber tow, High-temperature properties, Resistive heating, Temperature feedback, Concurrent function, Non-ohmic

## Abstract

Measuring temperature through carbon fiber reinforced plastics requires an implanted contact-based temperature sensor during resistive heating. Implanting the sensor brings about considerable complications in the heat-joining of soft biocompatible Carbon Fiber Reinforced Plastics (CFRPs). In this paper, the concurrent temperature-dependent Electrical Resistance (ER) behavior of Carbon Fiber (CF) tow along with resistive heating is introduced. The temperature feedback from CF tow was investigated in the range of 60–200 °C in the room condition. The process is characterized by high nonlinearity due to complex mode of heat loss, orthotropic and semi-conductive nature of CF, resistivity of contacts, gas-moisture adsorption and ambient changes. In such conditions, experiments were conducted to study the Current-Voltage (I–V), ER-time and ER-temperature in steady-state and transient modes. I–V relationship was non-ohmic and ER-temperature relationship showed negative temperature coefficient at temperatures above 60 °C. Exponential behavior similar to that of thermistors was identified in ER-temperature relationship. The relationship is expressed by Hoge-quartic model, $\frac{1}{T}=a+b\left(\ln\;R_{\left(T\right)}\right)+c{\left(\ln\;R_{\left(T\right)}\right)}^{2}+d{\left(\ln\;R_{\left(T\right)}\right)}^{3}+e{\left(\ln\;R_{\left(T\right)}\right)}^{4}$, showing the best fit among the conventional calibration equations of thermistor. The reversibility of ER-temperature relationship with maximum error of 16.4 °C was observed. The repeatability of the relationship shows the CF viability of providing concurrent temperature feedback during high-current Joule heating in the room condition.

## 1 Introduction

A multifunctional material with low electrical conductivity like Carbon Fiber (CF) enables the material to sense the temperature, measure the humidity, and store the electrical energy (Forintos and Czigany, 2019; Johannisson et al., 2018). Besides outstanding mechanical nature (Park, 2015), CF has shown great viability as Joule-heating element in soft actuators to control stiffness (Hoang et al., 2021), polymer matrix heating (Fukuda, 1994; Tridech et al., 2013) and thermoplastic joining (Eveno and Gillespie, 1988; McKnight et al., 1997; Villegas et al., 2013). X-ray translucency and biocompatibility of CF (Petersen, 2016) increases the potential of this low-density non-metallic conductive fiber for metallic-allergy-free medical devices (Steinberg et al., 2013; Tarallo et al., 2020) and electromagnetic shielding (Chung, 2004) near Magnetic Resonance Imaging (MRI) scanner (Lee et al., 2018).

Implanting a temperature sensor like a thermocouple into Carbon Fiber Reinforced Plastics (CFRPs) or attaching to carbon fiber tow as a thermal feedback has some hindrances such as design, structure strength, cost, and manufacturing (Wang et al., 2004), especially when dealing with large area. Athanasopoulos et al. utilized a thermal camera to track temperature, while it could only provide an average measurement with time lag and minimum error of ±5 °C at which heating element is implanted through matrix (Athanasopoulos and Kostopoulos, 2012). Temperature-dependent resistance of CF has the potentiality to solve the problem. CF as part of a composite structure can be a light-weight heating element and temperature sensor without disturbing the structural homogeneity and compromising measurement and design integrity.

Majority of conventional studies focused on CF Joule heating or resistive heating behavior for joining feasibility (Fukuda, 1994), quality and scales of thermoplastic welding (McKnight et al., 1997), and melting over glass transition temperature (Tridech et al., 2013). In these studies, thermocouples were used to control the target temperature.

For clarification, according to the heat distribution model in steady state with internal heat generation shown in Eq. (1), the relationship between Joule heating and temperature in an orthotropic material like CF in the room condition is highly nonlinear. In this equation, Thomson’s thermoelectrical effect has been neglected compared with Joule effect.

(1) $$U.J=-\nabla .K_{CF}\nabla T+{\dot{Q}}_{Radi}+{\dot{Q}}_{Condu}+{\dot{Q}}_{Conve}$$

where U is electric field vector; J is current density vector per unit volume; K_CF_ is thermal conductivity of CF per unit volume; ∇T is temperature gradient; ${\dot{Q}}_{Radi},{\dot{\;Q}}_{Condu},and{\dot{\;Q}}_{Conve}$ are heat loss rate per unit area by radiation, conduction, and convection, respectively. The nonlinearity of this equation is mainly due to the relationship of partial differential term as $\nabla .K_{CF}\nabla T$ and forth order radiation heat loss shown in Eq. (2)which elaborates the heat losses.

(2) $${\dot{Q}}_{Radi}+{\dot{Q}}_{Condu}+{\dot{Q}}_{Conve}=\varepsilon_{\left(T\right)}\sigma A\left(T^{4}-T_{\infty }^{4}\right)+K_{CF}A\left(T-T_{C}\right)+h_{conve}A\left(T-T_{\infty }\right)$$

where $\varepsilon_{\left(T\right)}$ is thermal emissivity; σ is Stefan–Boltzmann constant; A is surface area; $T_{\infty }$ is the surrounding temperature; and, $h_{conve}$ is the convection coefficient. Previous studies attempted to reduce this nonlinearity through neglecting and simplifying terms. Wang et al. focused on only temperature sensing characteristics of CF (Wang et al., 2004). The study investigated the thermistor and thermocouple characteristics of similar and dissimilar CF composites. In this research, an external heat source controlled and raised the temperature at the target areas in the CFRP to reveal temperature-dependent behavior up to 200 °C. The partial differential, radiation and convection heat loss and internal heat generation terms were neglected. These were mainly because of utilizing the external heating source, thermal uniformity and supplied CF with low voltage-current which caused dramatic simplifications and linear resistance-temperature relationship in the study of thermistor behavior. Similarly, Yang et al. (2007) used external heater and thermocouple to investigate the Electrical Resistance (ER) temperature-dependent sensitivity and behaviors of hybrid and similar CF in CFRPs. The linear relationship of ER-temperature and its reversibility resulted in the studied range of 0–70 °C. Applied current intensity was kept as small as 15 mA to avoid self-heating which causes partial differential and heat loss terms to be neglected. Low intensity current also diminished the contact heating at the electrode interfaces. To model temperature with respect to time and electric potential, Athanasopoulos et al. (2012) investigated the resistivity-temperature relationship in polyacrylonitrile-based CF tows. The relationship was studied under insulated high vacuum pressure and away from ambient air. The author also neglected the generated heat at the CF-electrodes interface due to the use of silver paste, while current has been introduced by two-probe technique. In the aforementioned processes, partial differential term and the heat losses through conduction and convection modes were considered negligible. This simplified the heat transfer equation significantly; however, it limited the applications of the resistive heating CF to atmosphere-free ambient.

There is limited research on considering multi-functional properties of CF as concurrent high-intensity-current resistive heating and self-temperature sensing in the room condition. In order to attain temperature feedback generated by resistive-heated CF, it is of application and fabrication interests to use temperature-dependent property of CF to be a temperature sensor while CF is part of the reinforcement. In this paper, the resistive behaviors of high-strength carbon fiber tow are investigated through the I–V and ER-temperature relationships during Joule heating. Rising Current Test (RCT) and Cyclic Current Test (CCT) were conducted to investigate the relationships via a dedicated set-up. The set-up works based on four-probe technique to electrify CF. In this device, CF-to-electrode fast connectors and a tensioner have been provided to improve CF-electrode contact and to straighten specimens, respectively. The specimens were electrified with increasing the current intensity in RCT and with the particular current in CCT test in the set-up. For practical purposes, specimens were slightly pressed between thick black rubber parts during Joule heating. In this technique, all the heat losses, shown in Eq. (2), were assumed to be united together to self-heat the specimen predominantly. Regardless of the nonlinearities, the pressed CF behaved as a self-heating self-sensing unit. ER and ER-temperature behaviors of this unit should be identified in terms of temperature sensing while heat is generated via resistive heating. During the whole process, specimens were exposed to the room air.

Experimental results indicated that the current-voltage relationship had a slight non-linearity in a wide temperature and current range of 60–200 °C and 0.8–3 A, respectively. The ER-T relationship has an exponential behavior similar to that of thermistor. Among the governing equations on thermistors, Hoge-quartic equation showed adequate fit to the relationship in the range, as well as a good fit for the particular current of 2.9A at which specimen reaches 160 °C. The relationship is slightly reversible from the first to the second cycle with maximum error of 49.2 °C, but CF specimens would show a better maximum error of 16.4 °C from the second cycle onwards. The relationship varied in different specimens which can be attributed to minor thermal history, pattern dissimilarity, ambient changes, and moisture-gas adsorption at the temperature range.

## 2 Motivation

Developing a novel open-source printable modular soft endoscope is the ultimate goal of this study in order to have a low-cost disposable endoscope. While Endoscopy is known as one of the most accurate diagnosis and treatment techniques in many gastrointestinal tract diseases, the cost and availability concerns are still to be resolved. One solution is to break down the disposable part of the endoscope such as body and steerable head into short printable modules. The soft biocompatible plastic modules have a tubular structure and hollow channels longitudinally and through the tubular body. These structural features and the necessity of joining them to a single continuum soft body rule out conventional joining techniques. Utilizing a CFRP in form of a heating socket called ‘electrofuser’ can join and seal the tubular modules together. The CF within the reinforced plastic heats up to a target temperature to interfuse the tubular modules. A durable, resilient and sealed join involves the control of the generated heat at the interface. Feedback received from resistance-temperature relationship of CF can replace the use of implanted temperature sensor. The primary advantages would be compactness, cost-effectiveness, design simplicity, and structural homogeneity. This study contributes to the applications where weight, non-metallic non-magnetic feature, biocompatibility, out-of-autoclave curing (Liu et al., 2019), and relatively accurate control of temperature in joining of CFRPs are the main concerns.

## 3 Methodology

### 3.1 Materials

Unidirectional high strength CF tow specimens were collected from a T300 12K bidirectional textile with a 7 μm-fiber diameter. The Specimens dimensions were (7.5 ± 0.6) W × (130 ± 5) L × (0.7 ± 0.05) T (mm). The high strength carbon fiber has been chosen as it showed a linear temperature-resistance relationship (Yang et al., 2007), heat capacity, and very low dependency of thermal conductivity to temperature (Villière et al., 2013; Yamane et al., 2000). The CF mechanical and functional properties are listed in Table 1. A total of 50 specimens were taken from which 30 specimens were selected on the basis of in-range width 7.5 ± 0.6 mm, minimal fiber damage, and discontinuity.

**Table 1:** Carbon fiber mechanical and functional properties provided by manufacturer.

Properties	Value (unit)
Tensile Strength	3.53 GPa
Tensile Modulus	230 GPa
Specific Heat	0.19 Cal/g·°C
Thermal Conductivity	0.025 Cal/cm·s·°C
Electrical Resistivity	1.7 × 10^-3^ Ω·cm
Chemical Composition	93% Carbon
Na + K	<50 ppm
Filament Diameter	7um

**Figure 1:**
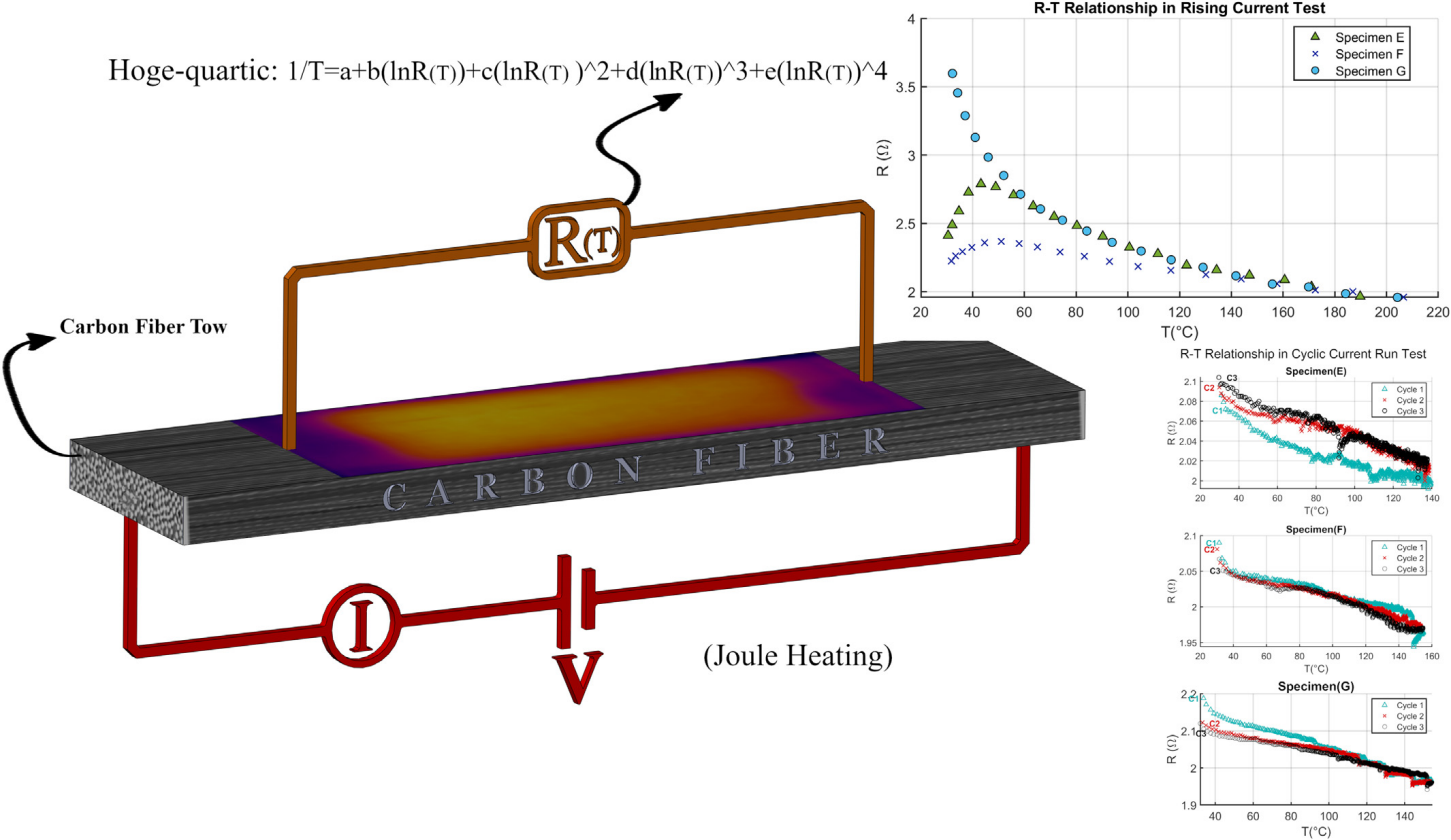
Graphical representation of this study’s processes.

The experimental set-up was used to introduce the current, temperature measurement, and pre-tensioning of CF shown in Figure 1. The primary components consisted of the power supply (Keysight-U8030), FLUKE 116 multimeter, and data acquisition (DAQ) (Power Meter HIOKI-PW3336), including two K-type thermocouples and a PC. The accuracy of current, voltage, resistance, and temperature measurement with the Power Meter was 0.001V, 0.0001A, and 0.1 °C, respectively. Based on pre-tests, the approximate maximum required DC voltage, current and power were 6 (V), 3(A), and about 20 (Watt), respectively, to reach the temperature of about 160 °C in CF with actual length of 40 mm within 10 min. Based on the literature (Kurokawa et al., 2008; Stopforth et al., 2017; Salemi et al., 2006; Spröwitz et al., 2010; Zhang et al., 2016), this length is found to be practical.

### 3.2 Methods

In CF resistive heating, the specimen heats up when current is being introduced to the ends. However, contacting metal electrodes to CF is characterized by contact resistance which has been a challenge to many studies (Carlson et al., 2011; Enoki et al., 2012; Hayes et al., 2015; Shirshova et al., 2013; Wu et al., 2006; Yang et al., 2007, 2009; Zantout and Zhupanska, 2010) in terms of insufficient or unstable contact, oxidation sensitivity, and complexity of preparing a secure interface. These studies used different configurations depending on their application to introduce current to CF. A biomimetic rod and socket mechanism have been used to boost the contact area and connectivity of the electrodes and CF, especially when a fast connection was demanded. Conductive paste like sliver paste has not been used as it increases the complexity of process, cost and incompatibility to the future applications of this study.

For further clarification, in all experiments, CF specimens pre-tensioning started 24 h before each experiment with a spring force of about 10 N to minimize local heating and maximize the straightness of the CF specimens. The ambient temperature was kept at 25 ± 0.3 °C during all tests and the relative humidity was 50 ± 5% all the time. Also, the term ‘Carbon Fiber (CF)’ means continuous high strength carbon fiber tow in the next sections.

#### 3.2.1 Electrical resistance measurements: four-probe vs. two-probe technique

**Figure 2:**
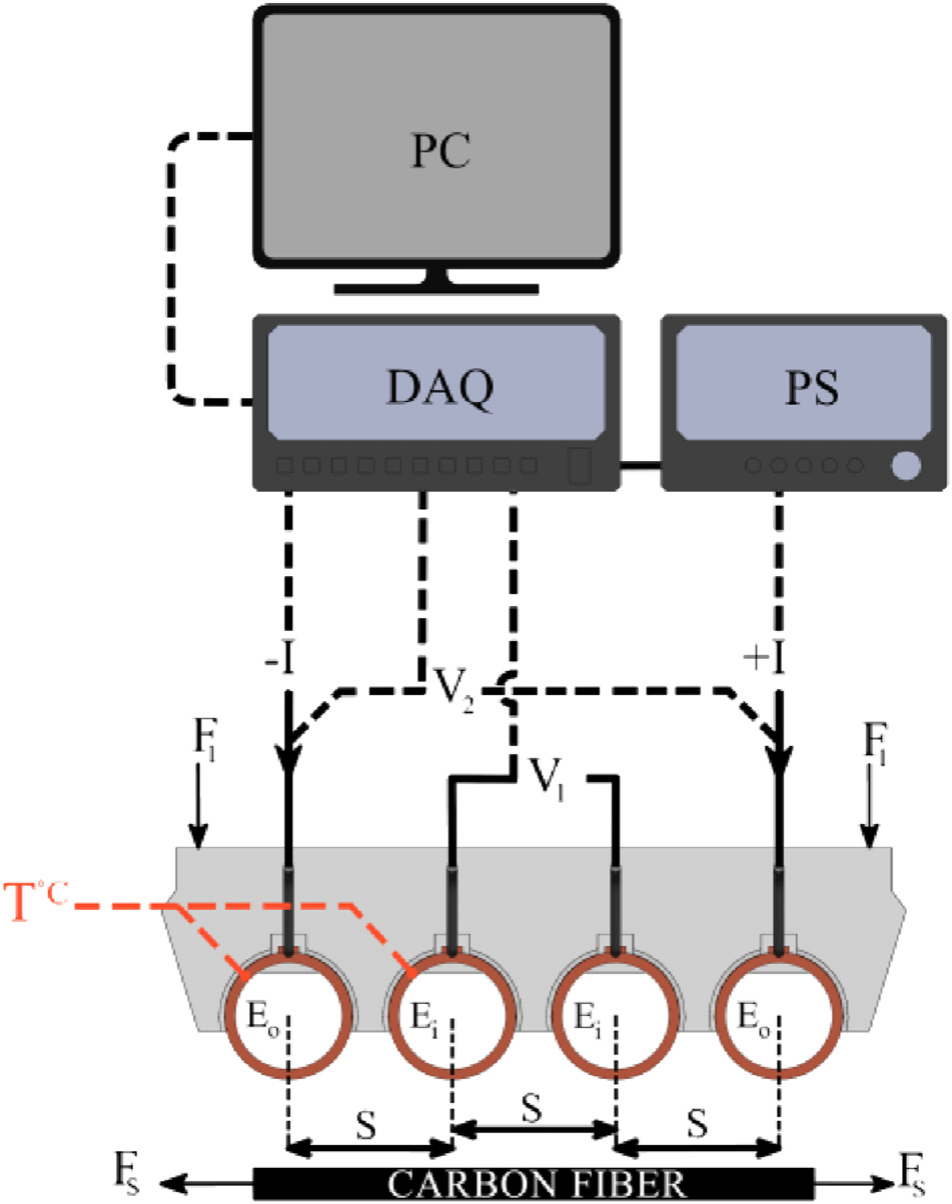
Four-probe and two-probe methods schematic. A computer (PC), data acquisition (DAQ), and power supply (PS) were used to measure the current (I), voltages (V_1_ and V_2_), and temperature (T °C) in outer electrodes (E_O_) and inner electrodes (E_i_). These electrodes were equidistantly installed with (S) distance.

The ER was measured by both two-probe and four-probe methods. Low ER, fibrous texture, and short length of specimen increase the importance of accurate resistance measurement. While the four-probe technique has more complexity and accuracy in situ, two-probe can significantly simplify the process of CF clamping, ER measuring during resistive heating, and implanting CF in matrix. The designed four-probe device is schematically shown in Figure 2. It consists of simple sub-components as equidistant-placed electrodes or probes and a semi-hard composite body. This body was designed to be self-adjustable on a larger area, instead of using spring to level the electrodes on a point. As a constant force of 26 ± 0.7 N (the ±0.7 N is due to the variation of electrode types and uncertainty of mass measurements), F_1_ was applied to the compartment to enhance the electrode-CF contact by adjusting and pressing the device during the ER measurement experiments as shown in Figure 2.

In contrast to other adjustable four-probe compartments or fixed attachment (Nishio et al., 2017), this mechanism can align the electrode with three-degree of freedom which can enhance the contact and rapid installment and measurement of CF. The width of the probes was slightly greater than that of the fabrics to ensure CF and electrodes were connected even in case of slight width variation or specimen deviation. Due to the good conductivity of copper, it has been widely used as electric clamps in CF studies in form of pure copper or tin-plated copper (Athanasopoulos and Kostopoulos, 2012; Athanasopoulos et al., 2012; Carlson et al., 2011; Wang et al., 2004; Wu et al., 2006; Yang et al., 2007, 2009); however, copper oxidation and great thermal conductivity can affect the accurate measurement. Two types of cylindrical copper electrodes were tested as cylindrical bar and pipe because of their great dimensional stability and excellent heat dissipation, respectively. Tin-plated electrodes were also provided to consider oxidation. To find a good electrode and electrical configuration fit to what this study requires, four sets of electrodes have been made for each four-probe device: Copper pipe set, copper tin-plated pipe set, copper bar, and tin-plated copper bar sets (Figure 2).

All sets measured the resistance of one specimen (A) three times to have comparable results in resistance. So, the four-probe compartment was placed on the specimen carefully and vertically in each test to minimize possible damage and fiber rearrangement. The current of 250 mA was introduced to the specimen A by the outer electrodes (E_O_) for 30 min. This current can heat up the CF specimen slightly higher than the room temperature. This current has been chosen to be at the best minimum range of our devices. The main electrical parameters were measured and recorded by DAQ as voltage (V_2_), current (I) of the outer probes, and voltage of inner probe (V_1_) (Figure 2). The temperature of one inner and outer electrodes (T), shown in Figure 2, was recorded manually.

Between each test, 30 min were given to the device and specimen to cool down naturally. Temperature at the electrodes was measured during experiment to observe the temperature fluctuation shown as T °C in Figure 2. Two-probe resistance was measured through the outer probes by R_(Two-probe)_ = V_1_/3I as resistance of length S. Four-probe resistance was recorded through the inner probe by R_(Four-probe)_ = V_2_/I of the same length. The selection of electrode and method for the accurate ER measurement in high density current tests has been based on the results obtained from these tests.

#### 3.2.2 Temperature measurement

Sheathed thermocouples were used in the present study because the other types of thermocouples such as foil-type can damage the fabric or cannot provide enough contact between CF and thermocouples. In the current study, it has been observed that applying force to the CF causes resistance reduction locally and, consequently, heat generation declines, which has been reported by Fukuda (1994). To reduce the heat loss and latency of temperature recording, the whole sheath surface was covered by polyimide. Eveno and Gillespie (1988) demonstrated that the heat distribution in CF is uniform when it is laminated. Whereas, CF in the room condition shows non-uniform heat distribution. It is of great importance to observe the extent to which the heat distribution can be non-uniform.

**Figure 3:**
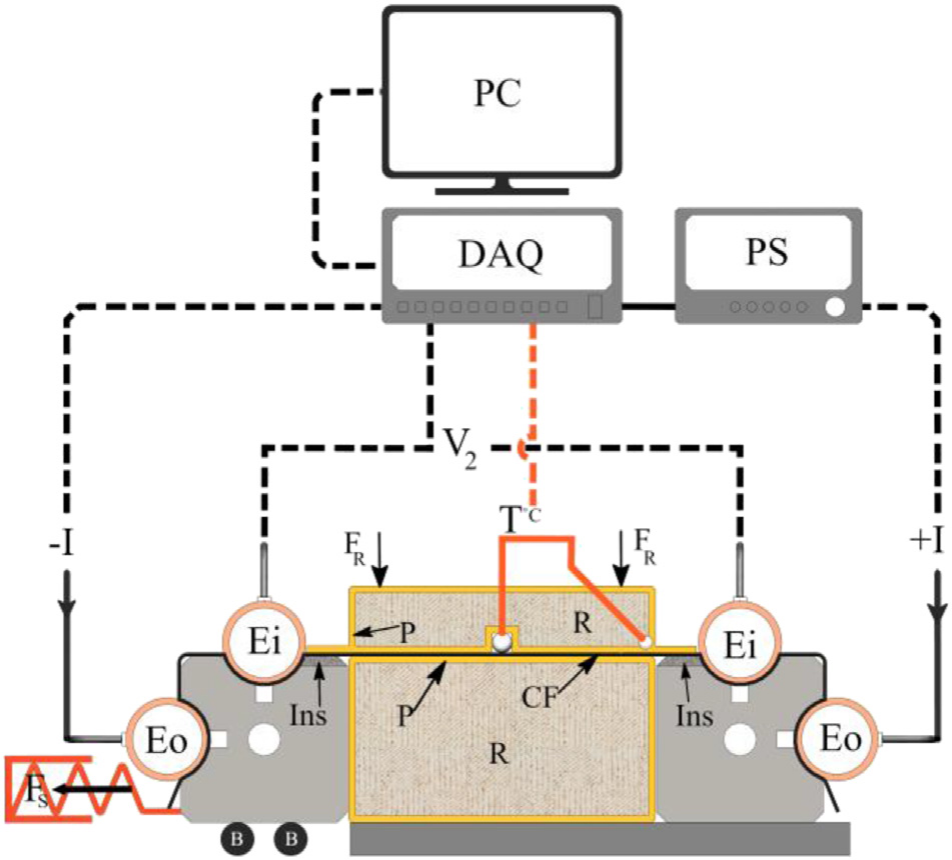
Schematic view of high current four-probe (HCFp). The Power Supply (PS) introduces current (I) to the outer electrodes (E_O_). The data acquisition (DAQ) unit measures the voltage of inner electrodes (Ei) and CF temperature (T), and records to the computer (PC), Applied Force (F_R_) to the rubber parts (R). Area near inner electrodes (Ei) is electrically and thermally shielded by an insulator (Ins). The tension spring applies Force (F_S_) to the electrodes holder which was installed over a linear Bearing (B).

On the other hand, examining the heat distribution in the fiber at different temperatures and areas could help us predict where the resistive heating would initiate; where the thermocouple can be installed correctly; and, what the temperature level is in near-electrode areas for material selection. To do so, a thermal camera Optris PI recorded the heat distribution of three specimens at different temperatures for 10 min without thermocouple. The specimens' temperatures were 45–70 °C at the currents of 0.5–0.9A, respectively. Thermal images were taken after thermal equilibrium and stability. The images have been processed in MATLAB R2020 software. To perform this test and further experiments, a testing device was manufactured to measure the parameters in higher currents and temperatures based on the resistance measuring results. This device is shown schematically in Figure 3 using the configuration of four-probe method resulted from ER measurement technique.

In the device, one pair of electrodes is movable as it is attached to a linear guide B shown in Figure 3. This guide gives one degree of freedom as translation to the electrodes and holds them in an accurate position and parallel to the electrodes fixed on the right-hand side shown in Figure 3. A tensioner spring was connected to the movable electrodes to apply the force of about 10 N. The distance between both ends of a specimen is measured and controlled by a gauge with the length of $40_{0}^{+0.2}$ mm. The effect of thermal strain on ER change has been neglected since the length of specimens was chosen short. Also, stress is infinitesimal due to change in the strain as a degree of freedom was given by the device. For further experiments with this device, the central thermocouple was attached to an accurate height adjustable linear bearing to maintain the consistency of contact, direction, and the force F_2_ as shown in Figure 3. Another thermocouple was installed through the rubber at the area near fixed-electrode. Both thermocouples, central and near-electrode thermocouples, were insulated electrically to avoid any possible short circuit between sheath and CF. This device is named as High Current Four-probe (HCFp) in the following sections.

#### 3.2.3 Temperature-resistance relationship

This section elaborates on the details of RCT and CCT tests in HCFp device through which the current-voltage relationship and ER-T relationships were studied. In RCT, the current was incrementally added, 0.15 A from 0.25 A to 3 A, and it was held for 15 min to reach a thermal equilibrium throughout all the fiber. Twelve specimens were tested and their current-voltage and ER-T relationships were extracted from logged data. In this test, the temperature range was 30–200 °C with corresponding current range of about 0.25–3 A while the specimens were exposed to the room air. This revealed the behavior of the CF in a wide range of current intensities.

**Figure 4:**
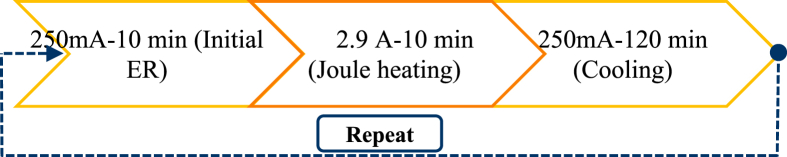
Sequential steps in CCT test to study the reversibility of ER-T relationship. In the first step, the initial ER was measured. In the second step, the CF specimens were heated with high current and then enough time was given to cool down naturally.

In CCT tests, the specimens were Joule heated cyclically. The current level and sequential steps are shown schematically in Figure 4.

When the specimens were heated up cyclically 10 times during pre-tests, CF showed relative reversibility and stability after the second cycle of resistive heating. So, majority of specimens passed three-cycle CCT. Since the electrical characteristics of CF can change in different currents, the objective of the CCT is to observe the ER-T behavior of a particular current 2.9 A and temperature 160 °C in a shorter time compared to RCT. This would be important in terms of controlling temperature in a fast-pace heating. In the first step, the current of 250 mA ran through one specimen for 10 min. In 10 min, the fiber could reach the temperature of about 30±2 °C at this current. In the next current level, in 10 min, CF reached the temperature of about 160 °C at the current of 2.9 A. After this, 120 min were given to the device and specimen to cool down naturally. In this test, one specimen is placed between two thermally and electrically non-conductive parts as shown in Figure 3. These parts are made of a hard-silicon rubber wrapped with polyimide to press the CF, R area and P layer shown in Figure 3, respectively. This experiment can show the electrified CF where it is reinforced (Athanasopoulos and Kostopoulos, 2012) or placed between two plastics for Joule heating (Athanasopoulos et al., 2010; Eveno and Gillespie, 1988; Fukuda, 1994) in the room condition. Although the rubber itself is an insulator, its phase might change in high temperatures which can lead to temperature change due to heat capacity variation. Therefore, a thick polyimide layer, as a relatively stable material in high temperature, was used to cover it. Static load of 26 ± 0.03 N, as F_R_ shown in Figure 3, was applied to the upper rubber-polyimide sandwich. This force would enhance the consistency of contact between the rubber parts and CF in all tests.

The upper part was designed to let the thermocouple reach and touch the CF for temperature measurement. Another thermocouple passes vertically through a hole in rubber to reach the CF near electrode. Relative softness of the rubber parts can compensate the surface unevenness. CF has no contact electrically in the area (Ins), shown in Figure 3. Using insulator at this area can significantly reduce the conductive heat transfer between CF and device. Yet, there is still heat residue that affects the heat distribution and temperature-resistance characteristics as initial condition and boundary condition of the test. To minimize the effect of heat residue between each test, 1-h time was given. Basically, the heat distribution is three-dimensional, i.e., longitudinal, transversal, and through thickness. It was assumed that CF is thin enough to be considered as a thin plate (da Gama et al., 2017) so the temperature gradient through thickness is zero. Due to the conduction mode of heat transfer between CF and polyimide in the given time, the heat was assumed to be distributed uniformly, transversally, and longitudinally in RCT. In this test, a central thermocouple would suffice this part of study. However, in the CCT, this assumption may not be valid and it has more of transient characteristics and the slope of ER-T graph may alter with a different thermocouple. So, one more thermocouple measures the temperature near-electrode. In pre-tests, the CCT test was repeated 10 times to study the behavior of CF specimens and to find the minimum number of cycles for heating to have a relative stable behavior by comparing the central and near-electrode thermocouples.

## 4 Results and discussion

### 4.1 Experiments

Resistive heating element is characterized by ER-T stability, durability, low oxidation and good heat distribution. Properties of a temperature sensor would be considered as chemical and electrical stability, longevity, reliability, and reversible ER. CF was used as both heating and temperature sensing unit since it has majority of aforementioned characteristics and properties. In this section, the results of 7 specimens are presented. Specimen A was used to compare four-probe, two-probe methods to design HCFp apparatus. Thermal images of specimens B-D improved the HCFp device configuration; revealed heat distribution; and, enriched mathematical assumptions. At last, the results of RCT and CCT tests of E-G specimens as three distinctive behaviors were shown.

### 4.2 Electrical Resistance measurements

#### 4.2.1 Four-probe, two-probe

**Figure 5:**
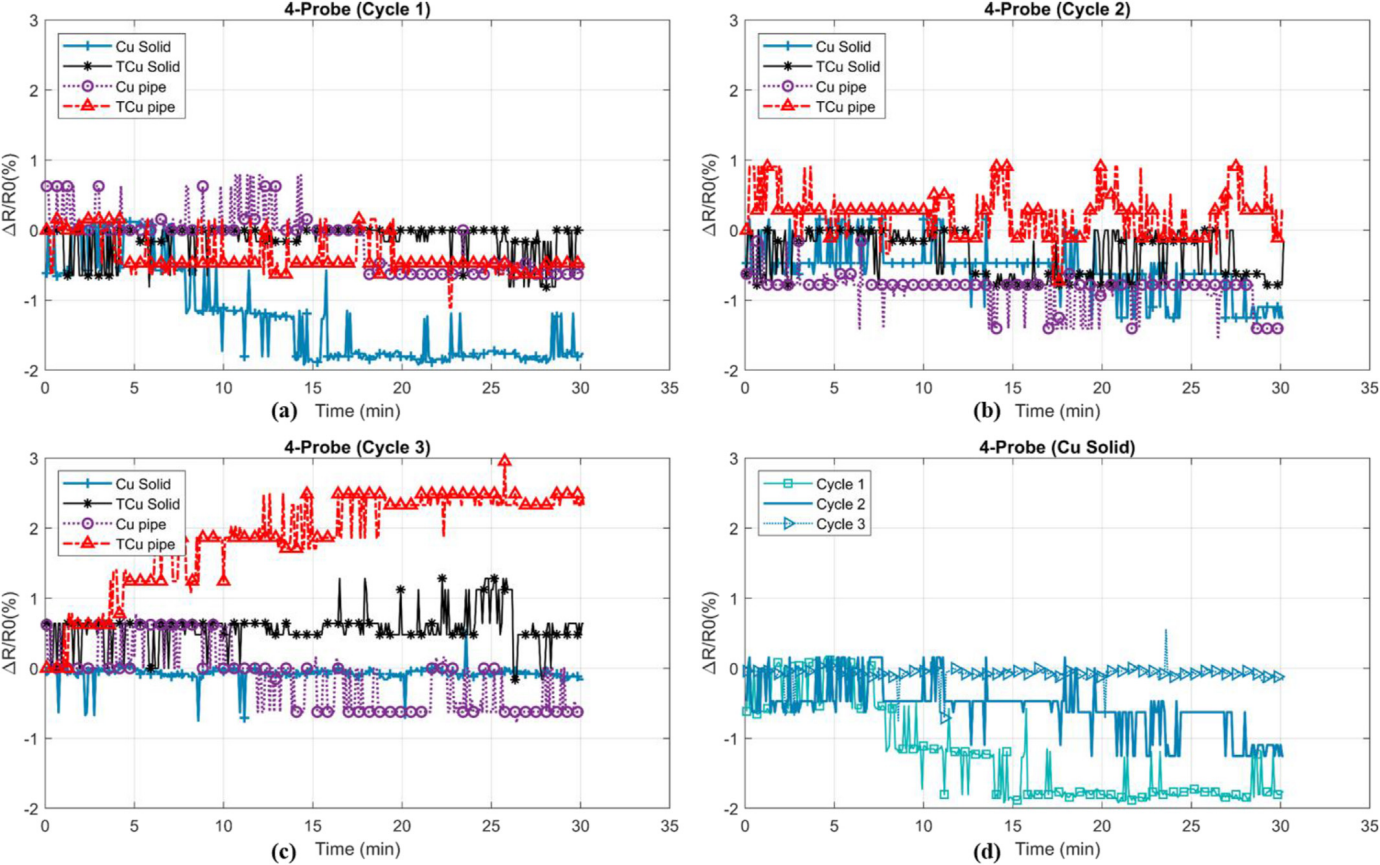
(a)–(c) The stability of relative ER with respect to time between different electrodes (d) Undulation comparison of preferable electrode as solid copper in three cycles.

The resistance measurements resulted from four-probe method as relative resistance (ΔR/R_0_) with respect to the time were obtained from four different sets of electrodes, shown in Figure 5.

Undulation of ΔR/R_0_ could denote the stability of temperature sensing with respect to time when both the room and the fiber temperature, humidity and current were kept constant. In Table 2, the initial ER (R0), initial Temperature (T0), and Standard Deviation (SD) Percentage (SDP) were compared. The SDP is a quantitative representation of the undulation, shown in Figure 2. Among four electrodes, the solid copper and tin-plated solid copper showed the highest stability with average of 0.39% and 0.57 % undulation.

**Table 2:** Measurement of four-probe resistance in different sets of electrodes as copper pipe (pipe Cu), copper tin-plated pipe (pipe TCu), solid copper (solid Cu), and tin-plated solid copper (Solid TCu) in three cycles (C1–C3). Standard Deviation (SD) represented undulation.

Electrode	Resistance		
	C1±SD %	C2± SD %	C3 ± SD %
Pipe Cu	1.44 ± 0.61	1.44 ± 0.431	1.46 ± 0.678
Pipe TCu	1.53 ± 0.40	1.50 ± 0.404	1.50 ± 1.221
Solid Cu	1.44 ± 1.01	1.44 ± 0.521	1.43 ± 0.185
Solid TCu	1.40 ± 0.31	1.45 ± 0.462	1.42 ± 0.386

Besides ambient changes like temperature and humidity, vibration can cause relative undulation in the results. All the tests were run in the Faraday Cage to reduce the effect of electromagnetic and electrical field. After few tests, some dark carbon residue was observed on the tin-plated electrodes. Natural softness of tin and imperfection of plating aid carbon leaves a stain on the electrodes. Since a negligible current (1 μA) passes through the inner electrode, the stain would have infinitesimal effect on the resistance measurement. So, the instability observed in Figure 5 can be attributed to geometrical imperfection of pipe electrode which may readjust during the test. So, the maximum resistance increase of 2.8% in this set of electrodes was observed. This test has been repeated 10 times, showing Negative Temperature Coefficient (NTC) or Positive Temperature Coefficient (PTC) behavior happens randomly. Both tin-plated and non-tin-plated solid electrodes showed a good consistency in the short term. But, these electrodes need a significant time to cool down compared to copper pipe electrodes.

Copper pipe and tin-plated copper pipe electrodes' manufacturing has more sensitivity in terms of geometry evenness and accuracy when compared to solid copper electrodes. Consequently, the inconsistency of the electrodes may cause CF-electrodes contact dissimilarity which resulted in different ER. Poor contacts can cause heat generation at contact area which elevates resultant ER there. Hence, the non-plated electrodes are preferred, in particular, solid electrodes to tin-plated electrodes as they showed higher consistency and stability in the recorded data due to geometry evenness, relative hardness, and free of oxidation property in the short term.

**Figure 6:**
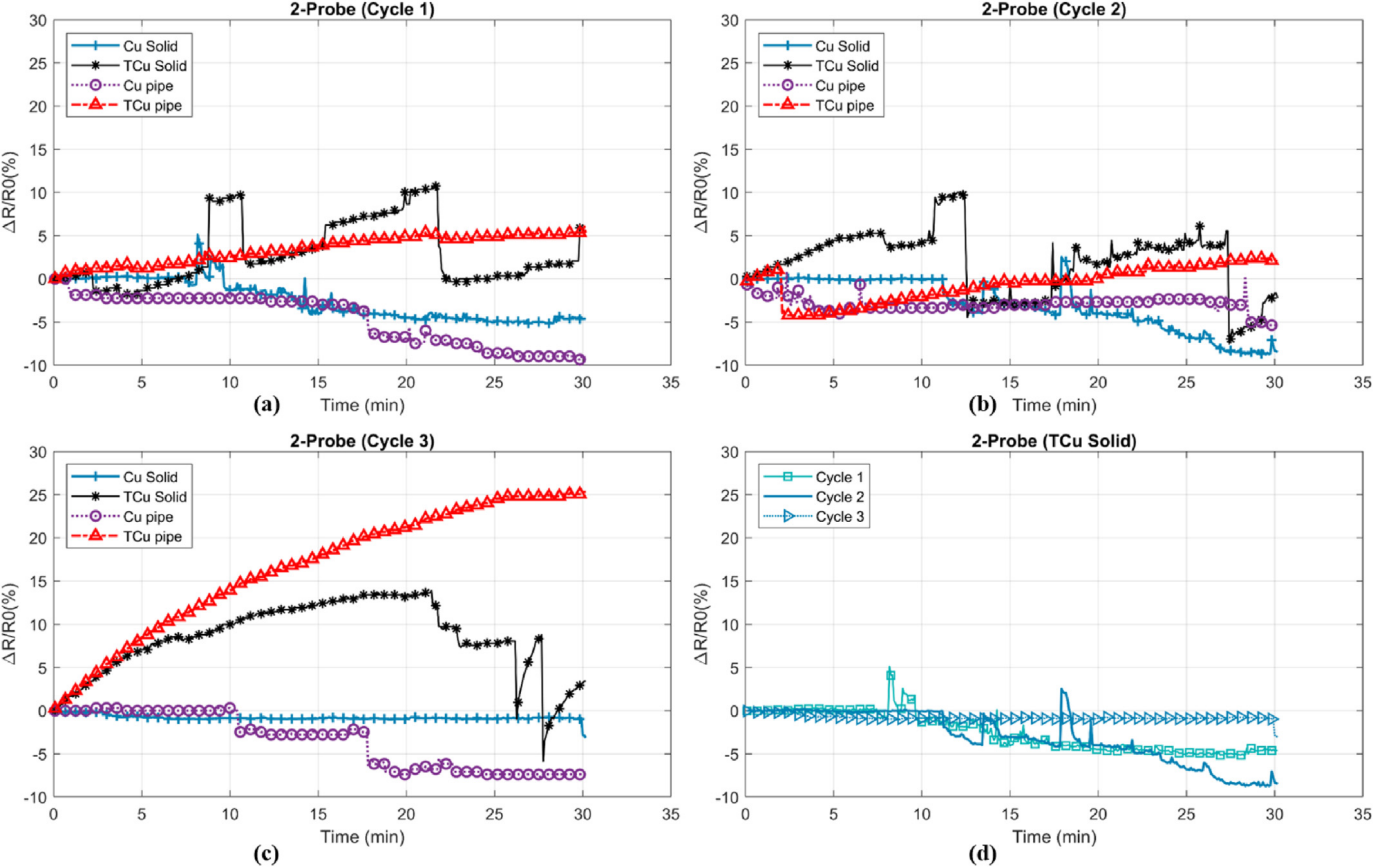
(a)–(c) The stability of relative ER with respect to time between different electrodes at constant current; (d) Undulation comparison of preferable electrode as tin-plated solid copper in three repetitions.

**Figure 7:**
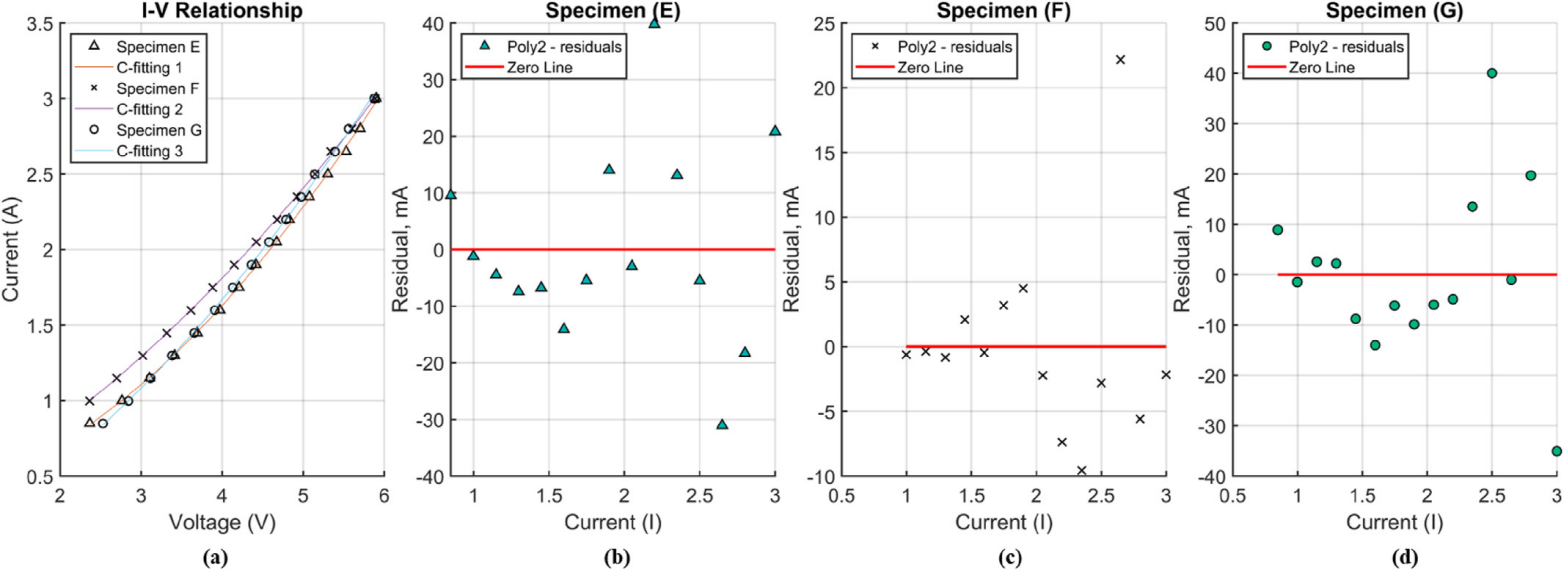
(a) Relationship between current and voltage I–V in different constant current levels of specimens E-G and quadratic fit (b)–(d) Residual plots of quadratic fit in specimens E-G.

The obtained two-probe resistance results as ΔR/R_0_ with regard to the time and the initial ER of four different sets of electrodes are shown in Figures 6 and 7, respectively, for the specimen.

Despite the four-probe, two-probe technique is characterized by instability, inconsistency, and offset. Tin-plated electrode sets showed that PTC and copper electrode sets often had NTC behavior. So, it could be concluded that two-probe method is highly affected by contact characteristics and add-up resistance of device and wires which cause unpredictability and instability in resistance measuring of the short length CF. In Table 3, the mean value of resistance, its SDP, and Resistance Offset Percentage (ROP) are presented.

**Table 3:** Measurement of two-probe resistance in different sets of electrodes as copper pipe (pipe Cu), copper tin-plated pipe (pipe TCu), solid copper (solid Cu), and tin-plated solid copper (Solid TCu) in three cycles (C1–C3), Standard Deviation (SD) relative resistance of two-probe and four-probe measurement (R_2_-R_4_/R_4_) %.

Electrode	Resistance		
	C1 ± SD % [((R2-R4)/R4) %]	C2 ± SD % [((R2-R4)/R4) %]	C3 ± SD % [((R2-R4)/R4) %]
Pipe Cu	1.96 ± 6.07 [36.1%]	2.18 ± 2.72 [51.4%]	2.38 ± 7.81 [63.0%]
Pipe TCu	3.21 ± 5.58 [109.8%]	2.85 ± 5.59 [90.0%]	3.14 ± 23.73 [109.3%]
Solid Cu	1.87 ± 4.35 [29.8%]	2.50 ± 7.84 [73.6%]	2.08 ± 0.83 [45.4%]
Solid TCu	2.20 ± 8.07 [57.0%]	2.78 ± 9.95 [91.7%]	3.34 ± 14.85 [135.2%]

ROP is defined as (R_2-probe_ − R_4-probe_/R_4-probe_) percentage comparing two-probe and four-probe ER measurements. The smaller the ROP value, the less offset the two-probe method showed. Among the probes, non-tin-plated electrodes showed less offset and resistance fluctuation. Further results showed that regardless of significant mean value offset of 45.4% in the non-plated solid copper, the minimum undulation of 0.83% can cause a significant error in the output of calibration equation. In the third cycle, carbon residue was observed on the outer electrodes. Significant SDP rise can be attributed to the carbon stain, high contact resistance, and dimensional imperfection. Using two-probe method as the measuring technique would produce significant error in this study and four-probe method would be the preferable method for high current resistance heating and ER measuring since contact resistance has a very negligible effect. Therefore, in the HCFp, non-plated solid copper electrodes have been used.

#### 4.2.2 Current-voltage relationship

Figure 7 shows the current-voltage (I–V) relationship in specimens E-G as a result of RCT in the range of 60–200 °C.

In Figure 7, a minor non-linear behavior is seen in the relationship. Table 4 indicates the coefficients of quadratic equations in the specimens (i.e., ${V=r_{0}+r_{1}I+r_{2}I}^{2}$), where r_0_, r_1_, and r_2_ are constants. Some contributors of I–V quadratic relationship would be nonlinearity of heat loss which causes nonlinearity of resistance and variation of electrical characteristics at different currents (Owston, 1970). The constants of these fitting curves are estimated using linear least squares method. Squared correlations coefficient (ordinary R-Squared) of all fitting curves are greater than 0.999. The residual plot per each specimen is shown in Figure 7, specimen E to specimen G. The residual is defined by Eq. (3). The pattern denotes a random distribution (Montgomery and Runger, 2013) of quadratic model in the specimens.

(3) $${Resiual}_{i}={Data}_{i}-{Fitted\;Value}_{i}$$

**Table 4:** Estimated coefficient and squared correlations coefficient (R-squared) of I–V relationship for specimens E-G.

Parameters	Specimen		
	E	F	G
*r*_*0*_	0.3016	0.1910	-0.1065
*r*_*1*_	0.0749	0.2497	0.2520
*r*_*2*_	0.0643	0.0387	0.0480
R-Squared	0.999	0.999	0.999

### 4.3 Temperature measurement

**Figure 8:**
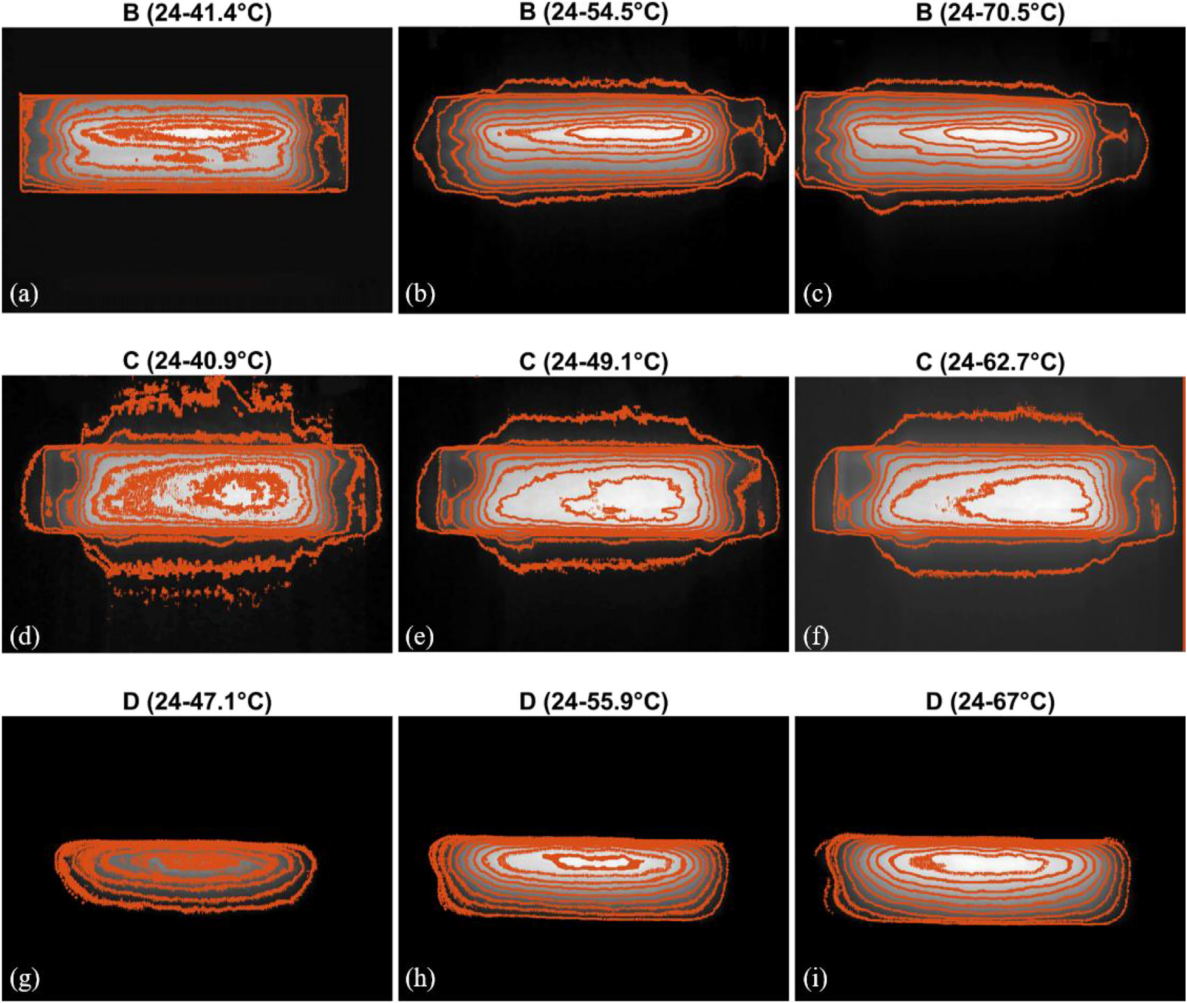
Heat distribution on three specimens (Sample, Temperature range °C, Current A): (a) B, 24–41.4 °C, 0.5 A, (b) B, 24–54.5 °C, 0.7 A, (c) B, 24–70.5 °C, 0.9 A, (d) C, 24–40.9 °C, 0.5 A, (e) C, 24–49.1 °C, 0.7 A, (f) C, 24–62.7 °C, 0.9 A, (g) D, 24−47.1 °C, 0.5 A (h) D, 24–55.9 °C, 0.7 A (i) D, 24–67 °C, 0.9 A. (g–i): The electrodes were not covered with insulator.

In Figure 8, isotherms are shown in specimens B-D when specimens are in the room air and before placing between polyimide parts. Maximum and minimum temperatures are noted on each image. The edges have the minimum temperature.

Non-homogeneity in the fibers in terms of shape and patterns like waviness, thermal history, nonlinear heat loss and imperfection of electrode-CF contact would lead to non-uniformity and dissimilarity of heat distribution (Athanasopoulos and Kostopoulos, 2012) and isotherms. Isothermal boundaries along with the fiber and hottest spots are not necessarily formed at the center transversally. So, thermocouples' contact at different areas can result in different values when the contact point is small. Contacting whole width of specimen with thermocouple could produce consistent results at the central and near-electrode areas perpendicular to the fiber direction, especially when it is pressed between two isolators (Figure 3) in the given time of 10–15 min. Uniformity of temperature was concluded from compared temperature of the central and near-electrode thermocouples while it is pressed between polyimide parts. In Figure 8, the area where the thermocouples are installed is demonstrated.

#### 4.3.1 Electrical Resistance-temperature relationship

**Figure 9:**
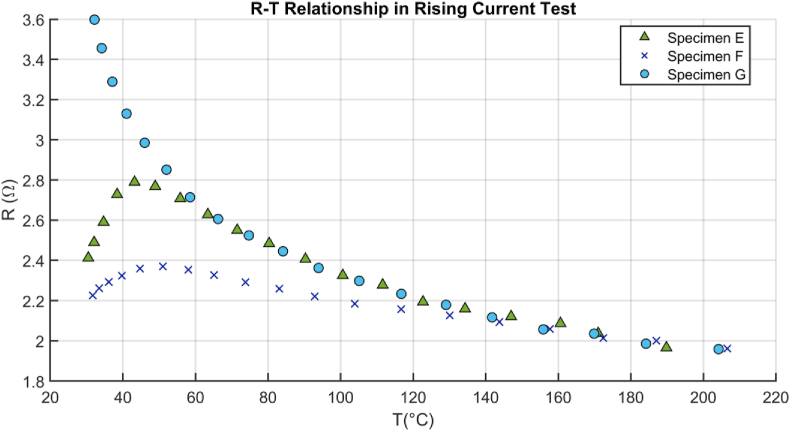
Exponential ER-T relationship of some specimens.

The results obtained from RCT in specimens E-G, in the range of 30–200 °C, show the ER-T relationship. ER-T plot showed a consistent exponential relationship at currents greater than 0.8A presented in Figure 9.

PTC characteristics in some specimens at temperature lower than 60 increase the uncertainty of the assumptions. Hence, this study is more focused on the temperature above this temperature which is near the glass-transition temperature of most of thermoplastics. The calibration equations to models ER-T relationship are presented as Eqs. (4), (5) and (6).

(4) $$\frac{1}{T}=\frac{\alpha }{1+\beta *\ln\;R_{\left(T\right)}}$$

where *α* and *β* are constants and linearly dependent on temperature

(5) $$\frac{1}{T}=a+b\left(\ln\;R_{\left(T\right)}\right)+c{\left(\ln\;R_{\left(T\right)}\right)}^{2}+d{\left(\ln\;R_{\left(T\right)}\right)}^{3}+e{\left(\ln\;R_{\left(T\right)}\right)}^{4}$$

(6) $$\frac{1}{T}=a+b\left(\ln\;R_{\left(T\right)}\right)+c{\left(\ln\;R_{\left(T\right)}\right)}^{2}+g{\left(\ln\;R_{\left(T\right)}\right)}^{-1}$$

where *a, b, c, d, e* and *g* are constants.

Eq. (4) known as the Basic equation is the rewritten form of two-constant exponential model (Chen, 2009) to define ER-temperature relationship based on two coefficients. This calibration equation provides a baseline with simple mathematical expression to be compared with the further equations. Eq. (5) enhances the fitting by adding terms of high order as quartic equation. Eq. (6) is another modified form of Hoge’s equation with square and rational ${\left(\ln\;R_{\left(T\right)}\right)}^{-1}$ terms (Hoge, 1988) which is called Hoge-rational in this study.

Non-linear least square method has been used for coefficient estimation of the equations in MATLAB software. This method showed functionality in calibration equation extraction of thermistors (Chen, 2009). In the estimations, 1/T was defined as dependent variable and R_(T)_ as independent. Estimated coefficients of the calibration equations have been listed in Table 5.

**Table 5:** Estimated coefficients and squared of ER-T for specimens E-G.

Model	Coefficients	E	F	G
Basic	*α*	0.0025	-0.8657	0.0032
	*β*	-0.8657	-1.0698	-0.7877
Hoge-Quartic	*a*	1.6548	7.0154	-0.2073
	*b*	-8.1765	-37.6822	1.0092
	*c*	15.0836	75.8810	-1.8265
	*d*	-12.2987	-67.9112	1.4721
	*e*	3.7556	22.8214	-0.4304
Hoge-Rational	*a*	0.8258	3.1256	-0.0630
	*b*	-1.0730	-4.3656	0.0498
	*c*	0.4789	2.0520	0.0098
	*g*	-0.2125	-0.7485	0.0202

Residual plot Eq. (3), which is to compare adequacy of fitting equations, and error of consecutive fitted curves Eq. (7), which is to find temperature measurement error between consecutive cycles, are the quantitative criteria.

(7) $$E_{j,j+1}={Fitted\;Value}_{j+1}-{Fitted\;Value}_{j}$$

where *E*_*j,j+1*_ is the error between the calibration equation of two consecutive cycles. The *fitted value* of a cycle (j+1) is subtracted from that of previous cycle (j). When it is not possible to present all residual plots or results of the plots are close to each other, the maximum (*Residual*^*max*^), minimum (*Residual*^*min*^) and average residuals (*Residual*^*ave*^) are compared. *Residual*^*ave*^ can be calculated with Eq. (8).

(8) |Residualave|=1n∑|Residuali|

where $\left|{Residual}_{i}\right|$ is the absolute of R*esidual*_*i*_ and *n* is the number of data.

**Figure 10:**
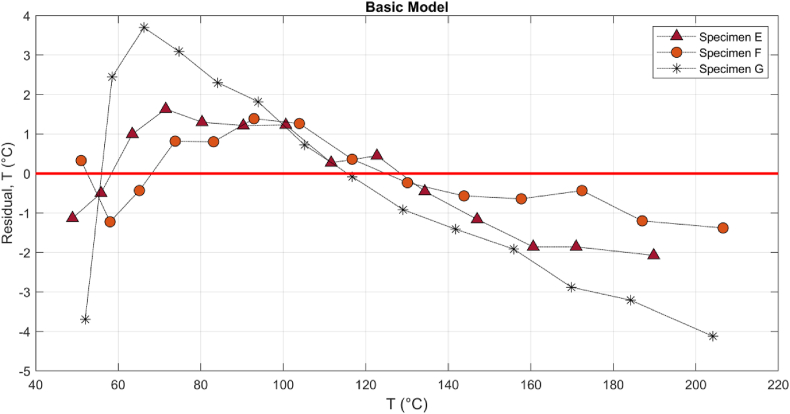
The residual plots of the basic model for three CF specimens E-G in RCT.

**Figure 11:**
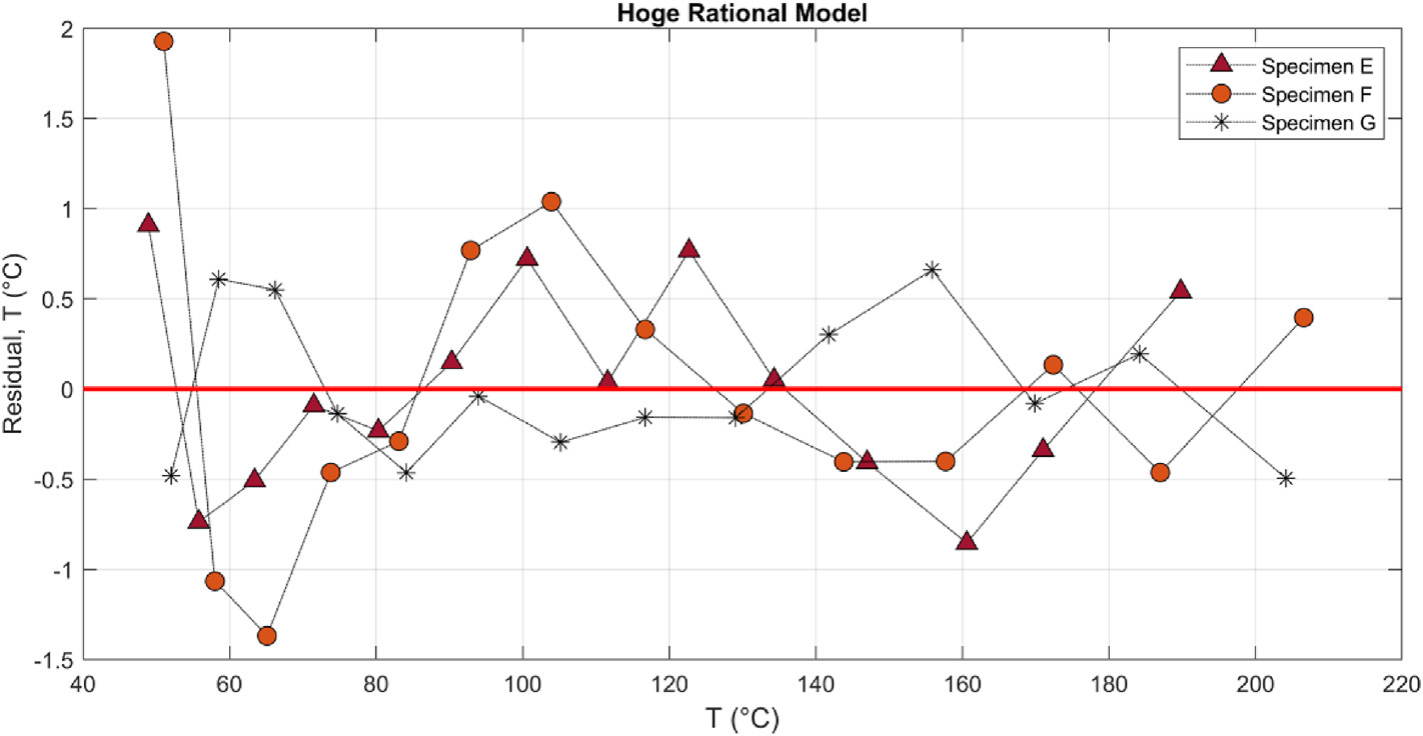
The residual plots of the Hoge-rational model for three CF specimens E-G in RCT.

Figures 10 and 11 show the residual plots of specimens E-G. Random distribution shows the adequacy of the basic, quartic and rational model introduced by Eqs. (4), (5) and (6), respectively.

**Figure 12:**
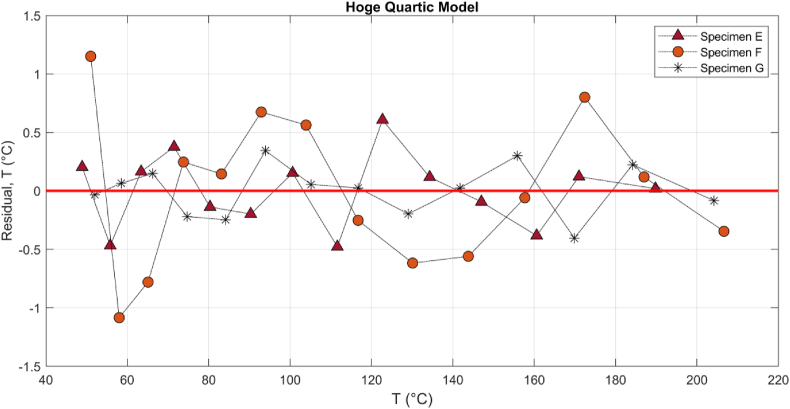
The residual plots of the Hoge-quartic model for three CF specimens E-G in RCT.

The minimum and maximum errors in the quartic, E_max_: 1.15 °C, E_min_: −1.08 °C, are 20 and 40 percent less than the rational model, E_max_: 1.92 °C, E_min_: −1.36 °C, respectively; however, the basic model, Eq. (4), showed a clear systematic pattern (Montgomery and Runger, 2013) with a significant residual error, E_max_: 3.69 °C, E_min_: −4.12 °C which is an indicator of the inadequacy of basic model. Figure 12 shows that different specimens E-G can have dissimilar ER-T relationship, but the characteristics may be more similar at higher current and temperature, particularly above 100 °C. The change in electrode-CF contact, pattern and number of fiber per specimens, and adsorption would alter the relationship.

### 4.4 Electrical resistance-temperature relationship reversibility

**Figure 13:**
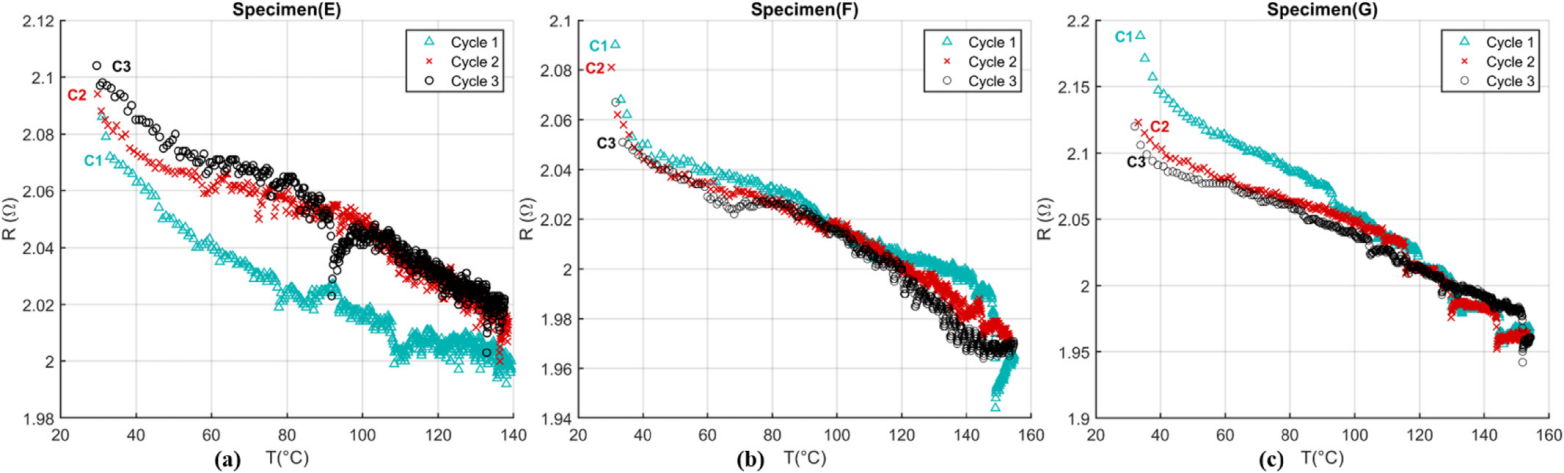
(a)–(c) ER-T relationship of specimens E-G while they are heated up resistively in CCT test. After each cycle, the relationship is comparable. Specimens reach the maximum temperature of about 160 °C.

The CCT test results are shown in Figure 13. In this test, the first cycles have shown distinct behavior. The plots would overlay in the second cycle onwards. Initial and overall ER in majority of specimens were reduced after the first cycle in the range of 2–10%.

To minimize the thermal residue which can change initial condition and consequently the initial resistance, enough time was given to the device and the specimens to cool down. The objective of this test was to study the ER-T relationship in a short time when a rapid heating is demanded to reach a target temperature when the state would be less steady compared to the RCT. Accordingly, the exponential curve shape and the slope of ER-T graph, shown in Figure 13, can be affected by the thermocouples type, heat dissipation latency, and heat capacity of CF. Similar calibration equations were studied for this relationship using Eqs. (4), (5) and (6). Their residual criteria compared the adequacy of the three models in Table 6.

**Table 6:** Criteria to compare three calibration equations for each cycle of resistive heating of CF.

Model	Error (°C)	E			F			G		
		Cycle 1	Cycle 2	Cycle 3	Cycle 1	Cycle 2	Cycle 3	Cycle 1	Cycle 2	Cycle 3
Basic	Residual^min^	-8.18	-6.01	-4.45	-3.36	-4.64	-4.68	-3.47	-4.57	-2.43
	Residual^max^	6.27	11.40	8.24	11.50	9.45	16.13	12.24	9.97	4.38
	|Residual|^ave^	1.18	1.60	0.99	1.977	1.18	1.73	1.05	1.45	0.69
Hoge-Quartic	Residual^min^	-4.74	-4.93	-4.02	-4.23	-4.11	-6.25	-4.24	-4.87	-3.77
	Residual^max^	6.34	10.25	7.60	9.14	5.56	12.01	9.30	3.43	2.45
	|Residual|^ave^	1.20	1.03	0.93	0.83	0.58	1.06	0.82	0.48	0.40
Hoge-Rational	Residual^min^	-4.74	-4.91	-4.39	-4.26	-4.32	-6.03	-4.29	-2.74	-2.57
	Residual^max^	6.43	10.07	7.77	9.18	5.82	11.82	9.37	3.58	3.66
	|Residual|^ave^	1.20	1.15	1.00	0.82	0.65	1.16	0.81	0.78	0.65

Among the models, the basic model showed significant error and inadequacy compared to the higher order models. The Hoge-quartic showed the best fit. While the Residual^max^ and Residual^min^ are slightly different between the Hoge-quartic and –rational, the |Residual|^ave^ in the Hoge-quartic is relatively better.

The rational model produces a considerable error when it comes to the reversibility analysis. To measure reversibility, the temperature deviation (*E*_*j, j+1*_) has been introduced in Eq. (7). In this equation, the predicted temperature of a cycle will be subtracted from that of previous cycle. So, the temperature error (*E*_*j, j+1*_) reveals to what extent the predicted temperature measurement is overlaid, especially between cycle two and three (E_2, 3_) when data show more consistency and reversibility. In this analysis, two adequate models are compared and presented in Table 7. As shown in Figure 13, the maximum gap of about 1.7% between the first cycle and the second cycle in the specimen (E) produces 49.2 °C error in the Hoge-quartic model which significantly drops between the second and the third cycles. In this specimen, Hoge-rational showed a marked error at 80.3 °C. The smaller the value of *E*_*j, j+1*_, the less error would be found between the predicted temperature of different cycles, and more reversibility for specimen.

**Table 7:** Fitted curves and squared correlations coefficient (R^2^) in I–V of specimens E-G.

Model	Criteria	S_1_		S_2_		S_3_	
		E_1,2_	E_2,3_	E_1,2_	E_2,2_	E_1,2_	E_2,3_
Hoge-quartic	T_error_ (°C)	49.2	12.6	14.4	16.4	14.4	16.4
Hoge-rational		80.3	15.5	28.4	26.7	28.4	26.7

## 5 Conclusion

CF was studied as thermal and thermistor element concurrently in the room air. ER-Temperature relationship in a wide temperature range, i.e., 60–200 °C, Current 0.8–3.0 A, and at a particular temperature, i.e., 160 °C, Current 2.9 A, was selected to study the concurrent and reversible behaviors. In the wide temperature range, the model adequacy is presented by the residual plot. In the particular temperature maximum, minimum and average residuals were compared. In the reversibility test, the relative error between two consecutive fitted equations provides the reversibility and model adequacy quantitatively. The results of this study indicate that ER-T showed NTC behavior in the wide temperature current range. This relationship was exponential and well fitted by Hoge-quartic equation ($\frac{1}{T}=a+b\left(\ln\;R_{\left(T\right)}\right)+c{\left(\ln\;R_{\left(T\right)}\right)}^{2}+d{\left(\ln\;R_{\left(T\right)}\right)}^{3}+e{\left(\ln\;R_{\left(T\right)}\right)}^{4}$) with maximum error of 1.1 °C. At particular temperature test, CF specimens showed exponential behavior in ER-T relationship. Similarly, Hoge-quartic equation was the best model. Although the residuals of the Hoge-quartic and Hoge-rational, $\frac{1}{T}=a+b\left(\ln\;R_{\left(T\right)}\right)+c{\left(\ln\;R_{\left(T\right)}\right)}^{2}+\frac{g}{\left(\ln\;R_{\left(T\right)}\right)}$, were somewhat similar, the Hoge-quartic was a slightly better model. In the reversibility analysis, the Hoge-quartic exhibited significantly less error. The maximum temperature errors of Hoge-quartic and Hoge-rational were 16.4 and 26.7 °C. Gas or moisture adsorption and complex mode of heat loss would cause variations in ER-T relationship reversibility. The CF showed the potentiality of providing temperature feedback during Joule heating concurrently for the application of CF as the electrofuser for resistive-heat joining of body modules in the modular soft endoscope in the room condition. Future studies could focus on microscopic aspects of low-high intensity current effects on ER change in CF and also testing the specimens in the presence of desiccant to reveal more of adsorption and current treatment characteristics.

## Declarations

### Author contribution statement

Danial Forouhar, BE, ME: Conceived and designed the experiments; Performed the experiments; Analyzed and interpreted the data; Contributed reagents, materials, analysis tools or data; Wrote the paper.

Jackrit Suthakorn, Ph.D.: Conceived and designed the experiments; Analyzed and interpreted the data; Contributed reagents, materials, analysis tools or data.

### Funding statement

This work was supported by Drug and Medical Supplies Delivery Mobile Robotic System (งป 032/2562) and Reinventing University, Mahidol Medical Robotics Platform (IO 864102063000).

### Data availability statement

Data included in article/supp. material/referenced in article.

### Declaration of interest’s statement

The authors declare no conflict of interest.

### Additional information

No additional information is available for this paper.

## References

[bib1] Athanasopoulos N., Kostopoulos V. (2012). Resistive heating of multidirectional and unidirectional dry carbon fibre preforms. Compos. Sci. Technol..

[bib2] Athanasopoulos N., Sikoutris D., Panidis T., Kostopoulos V. (2012). Numerical investigation and experimental verification of the Joule heating effect of polyacrylonitrile-based carbon fiber tows under high vacuum conditions. J. Compos. Mater..

[bib3] Athanasopoulos N., Sotiriadis G., Kostopoulos V. (2010).

[bib4] Carlson T., Ordéus D., Wysocki M., Asp L.J.P. (2011). CFRP structural capacitor materials for automotive applications. Rubber, Compos..

[bib5] Chen C. (2009). Evaluation of resistance–temperature calibration equations for NTC thermistors. Measurement.

[bib6] Chung D.D.L. (2004). Electrical applications of carbon materials. J. Mater. Sci..

[bib7] da Gama R.M.S., de Freitas Rachid F.B., Martins-Costa M.L. (2017). Mathematical modeling of heat transfer problems for thin plates with temperature-dependent conductivity. Heat Tran. Res..

[bib8] Enoki S., Iwamoto K., Harada R., Tanaka K., Katayama T. (2012).

[bib9] Eveno E.C., Gillespie J.R. (1988). Resistance welding of graphite polyetheretherketone composites: an experimental investigation. J. Thermoplast. Compos. Mater..

[bib10] Forintos N., Czigany T. (2019). Multifunctional application of carbon fiber reinforced polymer composites: electrical properties of the reinforcing carbon fibers–a short review. Compos. B Eng..

[bib11] Fukuda H. (1994). Processing of carbon fiber reinforced plastics by means of Joule heating. Adv. Compos. Mater..

[bib12] Hayes S.A., Lafferty A.D., Altinkurt G., Wilson P.R., Collinson M., Duchene P. (2015). Direct electrical cure of carbon fiber composites. Adv. Manuf. Polym. Compos. Sci..

[bib13] Hoang T.T., Quek J.J.S., Thai M.T., Phan P.T., Lovell N.H., Do T.N. (2021). Soft robotic fabric gripper with gecko adhesion and variable stiffness. Sensor Actuator Phys..

[bib14] Hoge H.J. (1988). Useful procedure in least squares, and tests of some equations for thermistors. Rev. Sci. Instrum..

[bib15] Johannisson W., Ihrner N., Zenkert D., Johansson M., Carlstedt D., Asp L.E., Sieland F. (2018). Multifunctional performance of a carbon fiber UD lamina electrode for structural batteries. Compos. Sci. Technol..

[bib16] Kurokawa H., Tomita K., Kamimura A., Kokaji S., Hasuo T., Murata S. (2008). Distributed self-reconfiguration of M-TRAN III modular robotic system. Int. J. Robot Res..

[bib17] Lee B.J., Watkins R.D., Chang C.M., Levin C.S. (2018). Low eddy current RF shielding enclosure designs for 3T MR applications. Magn. Reson. Med..

[bib18] Liu S., Li Y., Shen Y., Lu Y. (2019). Mechanical performance of carbon fiber/epoxy composites cured by self-resistance electric heating method. Int. J. Adv. Manuf. Technol..

[bib19] McKnight S.H., Holmes S.T., Gillespie J.W., Lambing C.L., Marinelli J.M. (1997). Scaling issues in resistance-welded thermoplastic composite joints. Adv. Polym. Technol..

[bib20] Montgomery D.C., Runger G.C. (2013).

[bib21] Nishio Y., Todoroki A., Mizutani Y., Suzuki Y. (2017). Piezoresistive effect of plain-weave CFRP fabric subjected to cyclic loading. Adv. Compos. Mater..

[bib22] Owston C. (1970). Electrical properties of single carbon fibres. J. Phys. Appl. Phys..

[bib23] Park S.-J. (2015).

[bib24] Petersen R. (2016). Carbon fiber biocompatibility for implants. Fibers (Basel).

[bib25] Stopforth R., Davrajh S., Althoefer K. (2017).

[bib26] Salemi B., Moll M., Shen W.-M. (2006).

[bib27] Shirshova N., Qian H., Shaffer M.S., Steinke J.H., Greenhalgh E.S., Curtis P.T., Kucernak A., Bismarck A. (2013). Structural composite supercapacitors. Compos. Part A: Appl. Sci. Manuf..

[bib28] Spröwitz A., Pouya S., Bonardi S., Van Den Kieboom J., Möckel R., Billard A., Dillenbourg P., Ijspeert A.J. (2010). Roombots: reconfigurable robots for adaptive furniture. IEEE Comput. Intell. Mag..

[bib29] Steinberg E.L., Rath E., Shlaifer A., Chechik O., Maman E., Salai M. (2013). Carbon fiber reinforced PEEK Optima—a composite material biomechanical properties and wear/debris characteristics of CF-PEEK composites for orthopedic trauma implants. J. Mech. Behav. Biomed. Mater..

[bib30] Tarallo L., Giorgini A., Novi M., Zambianchi F., Porcellini G., Catani F. (2020). Volar PEEK plate for distal radius fracture: analysis of adverse events. Eur. J. Orthop. Surg. Traumatol..

[bib31] Tridech C., Maples H.A., Robinson P., Bismarck A. (2013). High performance composites with active stiffness control. ACS Appl. Mater. Interfac..

[bib32] Villegas I.F., Moser L., Yousefpour A., Mitschang P., Bersee H.E. (2013). Process and performance evaluation of ultrasonic, induction and resistance welding of advanced thermoplastic composites. J. Thermoplast. Compos. Mater..

[bib33] Villière M., Lecointe D., Sobotka V., Boyard N., Delaunay D. (2013). Experimental determination and modeling of thermal conductivity tensor of carbon/epoxy composite. Compos. Appl. Sci. Manuf..

[bib34] Wang S., Kowalik D.P., Chung D. (2004). Self-sensing attained in carbon-fiber–polymer-matrix structural composites by using the interlaminar interface as a sensor. Smart Mater. Struct..

[bib35] Wu Z., Yang C., Tobe Y., Ye L., Harada T. (2006). Electrical and mechanical characterization of hybrid CFRP sheets. Compos. Sci. Technol..

[bib36] Yamane T., Katayama S., Todoki M., Hatta I. (2000). The measurements of thermal conductivity of carbon fibers. J. Wide Bandgap Mater..

[bib37] Yang C., Wu Z., Huang H. (2009).

[bib38] Yang C.Q., Wu Z.S., Huang H. (2007). Electrical properties of different types of carbon fiber reinforced plastics (CFRPs) and hybrid CFRPs. Carbon.

[bib39] Zantout A.E., Zhupanska O.I. (2010). On the electrical resistance of carbon fiber polymer matrix composites. Compos. Appl. Sci. Manuf..

[bib40] Zhang Y., Song G., Liu S., Qiao G., Zhang J., Sun H. (2016). A modular self-reconfigurable robot with enhanced locomotion performances: design, modeling, simulations, and experiments. J. Intell. Rob. Syst..

